# GLUE multimodal single cell data

**DOI:** 10.1093/pcmedi/pbad007

**Published:** 2023-03-20

**Authors:** Weizhong Li, Chaoyu Yan

**Affiliations:** Zhongshan School of Medicine, Sun Yat-sen University, Guangzhou 510080, China; Zhongshan School of Medicine, Sun Yat-sen University, Guangzhou 510080, China

## Abstract

A commentary on “Multi-omics single-cell data integration and regulatory inference with graph-linked embedding”

Single cell sequencing can obtain genetic information of large-scale cells at a single cell resolution. The technology has revolutionized life science research and accelerated discoveries in gene expression, cell development, immunology, and others. For instance, single cell transcriptomics has promoted our capability in presenting cell types and status. The outcome sequencing data from single cell technologies allows us to build a comprehensive reference atlas for human cells, enabling us to target complex diseases such as cancers. Different sequencing technology platforms can introduce multimodal single cell data. As new sequencing techniques keep emerging, the integration of single cell data from multiple modalities with distinct feature spaces becomes a major challenge. How to process multi-modality data has become a key obstacle for the single cell research community.

Single-cell omics data has the characteristics of high dimension and noise, as well as different feature spaces and systematic biases across different sequencing platforms. Current mainstream integration methods fall into three categories to handle the above characteristics. Category 1 is based on feature transformation. The multi-omics features are transformed directly into a same feature space, where the differences of omics data obtained from different sequencing techniques are rectified and the integrated cell representations are learned. Its representative algorithms include Seurat,^1^ Online iNMF,^2^ LIGER,^3^ and Harmony.^4^ The main drawback of this category of integration is causing significant information loss in the structure during feature transformation. Category 2 is based on manifold alignment, which conserves structural distances between cells in the original omics after integration. Methods in this category, such as Pamona,^5^ may misalign cell types due to lack of prior knowledge to guide the alignment direction. Category 3 is based on prior knowledge constraints. Methods in this category adopt prior knowledge between different omics based on the coupled matrix factorization algorithm, therefore can avoid information loss in the process of data transformation by Category 1 and correct the alignment direction by Category 2. Its representative algorithms are Coupled NMF^6^ and DC3.^7^ However, these tools can only integrate data from two omics modalities and their models are significantly complex, preventing them from running on datasets with over millions of cells.

Recently, Cao and Gao developed a computational method, named GLUE (graph-linked unified embedding),^8^ to fill the gap through modelling regulatory interactions across multiple omics layers. The GLUE framework combines the ideas of Category 1 and 3. It embraces a graphic coupling strategy to correlate different omics feature spaces using prior regulatory knowledge, and adopts adversarial learning to further minimize systematic disparities between omics in the transformation space. GLUE uses a knowledge-based graph called ‘guidance graph’ to model cross-layer regulatory interactions in linking feature spaces. The vertices in the graph represent the features of different layers while the edges illustrate the signed regulatory interactions. For example, an ATAC peak located near a promoter of a gene is often presumed to positively regulate the expression of the gene, so the edges between the two are denoted by positive weights. On the other hand, DNA methylation in a gene promoter is usually assumed to inhibit the gene expression, so the edges are denoted by negative weights.

GLUE generally follows the encoder-decoder structure, which is divided into the knowledge-based guidance graph part and the multi-omics integration part. The guidance graph leverages prior regulatory knowledge to correlate different omics spaces and guide the reconstruction of different omics in the multi-omics integration part. The graph neural network is applied to extract the low-dimensional representation of the guidance graph, and the inner product between the nodes after reconstruction is used to constrain the network training, so that the spatial similarity between the nodes in the low-dimensional representation of the graph can maintain the original spatial similarity. In the multi-omics integration part, GLUE follows the model structure of Cell BLAST.^9^ In the encoder, three VAEs are applied to reduce the dimension of each omics and integrate them into the same feature space. To maximally utilize the existing information, GLUE employs a linear method as the decoder, borrowing the concept of principal component analysis (PCA). In the low-dimensional space, the inner product of the graph matrix and each data matrix is implemented to reconstruct the integrated data with the same size as the input.

Cao and Gao further benchmarked GLUE against 10 popular unpaired multi-omics integration methods using three gold-standard datasets. GLUE achieved higher level of biology conservation and omics mixing against other methods across all benchmark datasets. Moreover, the authors applied GLUE to three application tasks: triple-omics integration (gene expression, chromatin accessibility, and DNA methylation), integrative regulatory inference, as well as multi-omics cell atlas construction. As a result, GLUE successfully revealed a shared manifold of cell states across three omics layers, explicitly modelled regulatory interactions, and effectively integrated gene expression and chromatin accessibility data into a unified human cell atlas with over millions of cells. With these advances, GLUE can help to create a comprehensive human cell atlas for understanding human health as well as providing insights into human diseases such as cancer, heart disease, respiratory disease, and in the long run, to promote precision medicine.^10^

Towards precision medicine, more and more different data modalities are emerging, such as scRibo-seq and scChIP-seq. We will soon have to face a new challenge in handling heterogeneous data of many more modalities and a significantly larger data size. Overcoming this obstacle will promote our research and applications in precision medicine. More powerful and smart tools are required for this purpose, and GLUE may be one of the significant steps towards that.
